# Efficient controlled release of cannabinoids loaded in γ-CD-MOFs and DPPC liposomes as novel delivery systems in oral health

**DOI:** 10.1007/s00604-023-05692-4

**Published:** 2023-03-09

**Authors:** Jorge Rodríguez-Martínez, María-Jesús Sánchez-Martín, Manuel Valiente

**Affiliations:** GTS Research Group, Department of Chemistry, Faculty of Science, Universitat Autònoma de Barcelona, 08193 Bellaterra, Spain

**Keywords:** γ-Cyclodextrin-MOFs (γ-CD-MOFs), Cannabidiol (CBD), DPPC liposomes (1,2-dipalmitoyl-sn-glycero-3-phosphocholine), Oral health, Synchrotron FTIR microspectroscopy

## Abstract

Olivetol (OLV), as a cannabidiol (CBD) analog, was incorporated in γ-cyclodextrin metal-organic frameworks (γ-CD-MOFs) and 1,2-dipalmitoyl-sn-glycero-3-phosphocholine (DPPC) liposomes as potential analgesic drug delivery systems (DDS) for dental hypersensitivity (DH) treatment. These DDS have been scarcely employed in oral health, being the first time in case of MOFs loaded with cannabinoids. *In vitro* experiments using bovine teeth were performed to verify if the drug is able to reach the dentin, where it can flow to the pulp tissues and exert its analgesic effect; enamel and dentin regions were analyzed by synchrotron radiation-based FTIR microspectroscopy. Principal component analysis (PCA) was used to process the spectroscopic data as a powerful chemometric tool, and it revealed a similar behavior in both regions. The studied DDS have been characterized by different techniques, and is was demonstrated that DDS is an efficient way to carry the drug through dental tissues without compromising their structure.

**Graphical Abstract:**

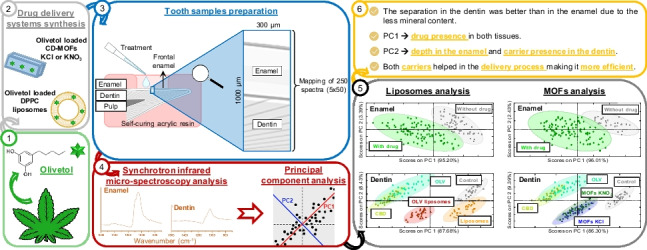

**Supplementary Information:**

The online version contains supplementary material available at 10.1007/s00604-023-05692-4.

## Introduction

Dental hypersensitivity (DH) could be defined as a short and sharp pain derived from exposed dentin in response to different stimuli [1]. Current strategies to avoid this sharp pain are not totally effective, and it is necessary to develop new treatments [2]. Cannabidiol (CBD) is the main nonpsychotropic component of *Cannabis sativa*, and it emerges as a promising candidate to avoid this pain since it can modulate receptors involved in the response caused by DH [3]. However, CBD shows low chemical stability and poor aqueous solubility [4]; the use of drug delivery systems (DDS) for its encapsulation or adsorption could overcome these disadvantages [5]. There are plenty of DDS; some of them applied in oral health [6]. Liposomes and metal-organic frameworks (MOFs) are well-known drug delivery systems and used in pharmaceutical formulations. Liposomes are spherical vesicles consisting of one or more phospholipid bilayers surrounding a water space that present the ability to encapsulate different substances; while lipophilic agents are entrapped in the bilayer, hydrophilic agents are enclosed in the aqueous core [7]. MOFs are porous crystalline materials consisting of metal ions or clusters linked to organic ligands by coordination bonds [8]. Due to all these characteristics, both systems have a wide range of application fields, such as gas storage, catalysis, or drug delivery [9–11]. In the case of medical applications, it is necessary to consider that the structures should be non-toxic for biological tissues. For liposomes, a quite studied phospholipid is 1,2-dipalmitoyl-sn-glycero-3-phosphocholine (DPPC), used as a model system to mimic the cell membrane [12]. Regarding MOFs, γ-cyclodextrin-MOFs (γ-CD-MOFs) have been reported as suitable materials in biomedicine due to its high biocompatibility since they are composed of γ-CD as organic ligand and usually potassium ions as metal linker [13]. Therefore, both systems present unique advantages for applications in encapsulation and release of cannabinoids. MOFs have been scarce studied as carriers of CBD, but some researchers have developed some liposomal formulations with CBD for biological and medical applications such as cancer treatment [14]. However, there are no specific reports about the use of CD-MOFs and DPPC liposomes loaded with CBD for oral health application, either other material used as CBD carriers for oral health; thus, our approach involves a novelty in this topic. CD-MOFs are biocompatible MOFs and they do not induce any toxicity up to 2000 μg/mL of concentration. And DPPC liposomes are well-known as DDS, and they have been already applied successfully in dentistry for other applications such as tooth whitening [15]. Besides, another important aspect for both systems is to verify whether scaling up the production of the formulation from the laboratory level to the industrial level is technically and economically possible. In these two cases, the authors have developed methodologies that can be scaled up.

Dental structure has been studied using different characterization techniques. Fourier Transform Infrared Spectroscopy (FTIR Analysis) is a very useful technique in dental research since it is able to study the tooth structure and its chemistry, organic and inorganic material, and their interactions, and it has been used to diagnose tooth structure alterations and pathologies [16]. Nevertheless, conventional vibrational spectroscopic methods are not always enough accurately to assess modifications in dental composition. Thus, the addition of a synchrotron radiation light source to FTIR technique (synchrotron radiation-based FTIR microspectroscopy, SR-μFTIR) provides the high intensity, spectral purity, and spatial and energy resolution necessary to study dental structure at micro- and nanolevels [17]. However, analysis of spectral data obtained is quite challenging due to the high number of spectra, the fact that many of the spectral bands are overlapping and small changes could not be appreciated using visual comparison. To solve these problems, chemometrics have been used, being principal component analysis (PCA) one of the most common applied in combination with FTIR spectroscopy since it can resolve small differences in the spectra and establish subgroups in the data [18].

Several researchers have already explored the large capabilities of the combination of synchrotron source infrared spectroscopy with chemometrics in dental field [19]. In this study, γ-CD-MOFs and DPPC liposomes containing olivetol (OLV) were synthesized and characterized as potential analgesic delivery systems for DH treatment. OLV was employed as an affordable CBD analog, since it is a precursor in cannabidiol biosynthesis, and they share part of the chemical structure [20]. To verify if the drug is able to cross the enamel and get into dentinal tubules, where it can flow to the pulp tissues and exert its analgesic effect, *in vitro* experiments using bovine teeth were developed. The primary component in the tooth is hydroxyapatite (HAP) that has well-defined bands in the infrared spectra (1600–700 cm^−1^), and the spectrum of CBD and olivetol has several absorption bands in the infrared region. Therefore, it would be possible to trace the penetration of the drug in the tooth structure using this technique coupling it to the synchrotron radiation source and using PCA for data processing. Furthermore, the penetration patterns according to the delivery system were evaluated and how the γ-CD-MOFs and DPPC liposomes help in the release process. Besides, the effect of the different treatments over the tooth was assessed based on the changes in the HAP band.

## Materials and methods

### Reagents

The following reagents were used: chloroform, potassium nitrate, potassium chloride, sodium azide, 2-[4-(2-hydroxyethyl)piperazin-1-yl]ethanesulfonic acid (HEPES), 1,2-dipalmitoyl-sn-glycero-3-phosphocholine (DPPC), cannabidiol, methanol, ethanol, polyethylene glycol 20000, olivetol, γ-cyclodextrin (98%), and MilliQ water. More details about these reagents can be found in the Electronic Supporting Material (ESM).

### Drug delivery system synthesis

A fast method using microwave irradiation was performed for γ-CD-MOF synthesis, and a cocrystallization method was used to load olivetol into these MOFs. This methodology has been described in a previous work [21]. DPPC liposomes were obtained by hydration and mechanical dispersion following a modified method previously described [15]. The complete description of these syntheses can be found in the ESM.

### Characterization techniques

Morphology and crystallinity of MOF samples were evaluated by Scanning Electron Microscopy (SEM) and X-ray powder diffraction (XRPD), respectively. Liposome dispersions were characterized by cryo-transmission electron microscopy (Cryo-TEM) and dynamic light scattering (DLS). All the details concerning the instruments and the measurements can be found in the ESM.

### Drug release and quantification

The quantification of the OLV content into the MOFs was performed following a methodology previously stablished [21]. For liposomes, the encapsulation of the drug was determined adapting a method previously described [7]. The drug encapsulation for each carrier was calculated following equation 1:

1 $$\mathrm{Drug\;encapsulation}\left(\mathrm{\%}\right)=\frac{\mathrm{Encapsulation\;OLV\;into\;the\;carrer\;}(\mathrm{mg})}{\mathrm{Total\;weight\;of\;OLV}-\mathrm{loaded carrier\;}(\mathrm{mg})}\cdot 100$$

All determinations were performed by triplicate.

The complete description of this experimental part can be found in the ESM.

### Tooth sample preparation

Bovine incisors were embedded in self-curing acrylic resin (Paladur, Germany) leaving the frontal enamel free of resin. Specimens were cut in half, and its lateral surface was polished down to 1 µm particle size. Then, teeth were treated with 0.1 mL of each treatment 3 times per day for 5 days (Table 1, Fig. S2.A). Since MOFs’ treatments are the only ones which are in powder, 1 mg of each MOF formulation was mixed with 15 µL of HEPES buffer and, then, was applied over the teeth. The complete description of this tooth sample preparation can be found in the ESM.

**Table 1:** List with the different treatments applied over the dental samples

Treatments’ list
Control (HEPES buffer)
Cannabidiol (10 mg/mL)
Olivetol (10 mg/mL)
DPPC liposomes
DPPC liposomes loaded with olivetol
γ-CD-MOFs KCl loaded with olivetol
γ-CD-MOFs KNO_3_ loaded with olivetol

### Synchrotron radiation-based FTIR microspectroscopy (SR-µFTIR) measurements

Specular reflectance spectra were acquired using FTIR microscope in the reflection mode at MIRAS beamline of ALBA synchrotron (Cerdanyola del Vallès, Spain). Hyperion 3000 microscope coupled to a Vertex 70 spectrometer (Bruker, Germany) and an HgCdTe (MCT) detector was used. A mapping of 300×1000 µm (wide x length) was made per each sample in the lateral surface from the outside of the enamel to the dentin. In each mapping, 250 spectra were measured having 5 spectra wide and 50 spectra length (Fig. S2.B). OPUS 7.5 (Bruker, Germany) was used to collect the spectra. All the details concerning the instruments and the measurements can be found in the ESM.

### Principal component analysis (PCA)

PCA was applied to process the SR-µFTIR data using PLS toolbox 8.2.1 (20559) for use with MATLAB 9.1 (R2016b). The big mapping per sample was limited to two smaller mappings, one in the inner enamel and another in the inner dentin. The wavenumber range was also limited from 1600 to 700 cm^−1^ where the main bands of HAP are located. Before PCA, mean centering preprocessing was applied to all the spectra. Besides, in each region of the tooth, enamel and dentin, liposomes, and MOF treatments were analyzed separately. All the details of the PCA can be found in the ESM.

## Results and discussion

### Drug delivery systems characterization and olivetol quantification

In order to evaluate the morphology and the crystal structure of the γ-CD-MOFs loaded with the drug, SEM and PXRD analysis were performed. The diffraction patterns of both γ-CD-MOFs KCl and γ-CD-MOFs KNO_3_ presented their peaks at the same 2θ angles (Fig. 1.1): 5.3°, 7.4°, 10.5°, 11.5°, 12.1°, 14.2°, 14.9°, 15.8°, and 16.7°. Peaks were quite narrow for both potassium salts, indicating high crystallinity, especially when KNO_3_ was employed since their peaks were sharper and more intense. These PXRD patterns have been already compared previously to the simulated references from the Crystalography Open Database (COD), including the JCPD card of the conventional CD-MOFs with the body-centered cubic space group *I*432 [21]. This recent publication showed the space group of these diffraction patterns matched with trigonal spaces groups when the metal linker was not delivered by potassium hydroxide [22]. This PXRD analysis agreed with SEM evaluation. The prism morphology of the crystals was the same for both potassium salts (Fig. 1.2). The size of these structures was quite heterogeneous, but most of them was around 2 to 50 µm. It could be explained due to the long incubation time to trigger the precipitation of the crystals that was 24 hours, since it is a key parameter in the kinetics that are involved in crystal growth [23]. Consequently, SEM and PXRD analysis showed crystalline CD-MOFs with prism shape, regardless of the potassium salt.

**Fig. 1:**
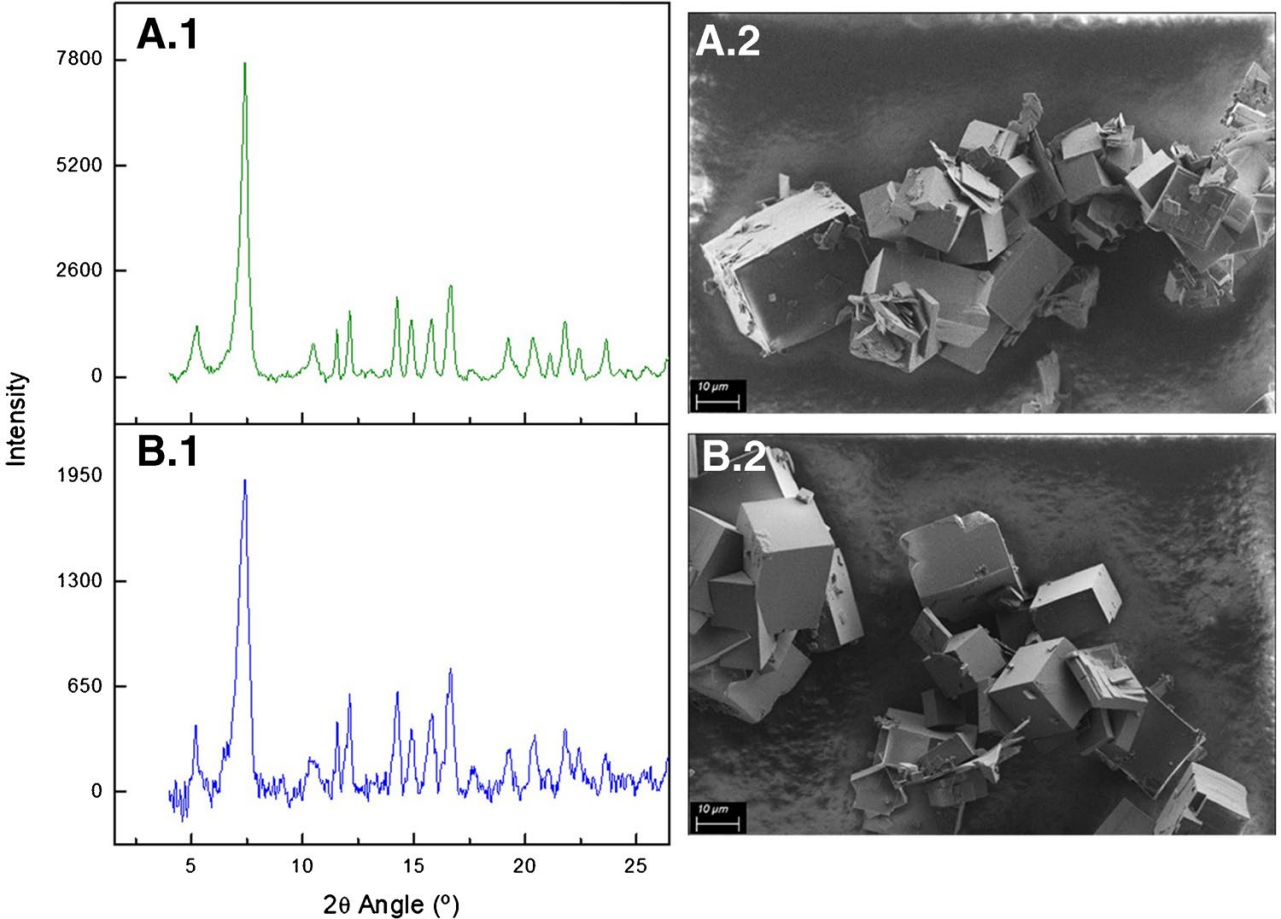
X-ray powder diffraction patterns (1) using Cu Kα anode (l = 1.5406 Å), tube voltage of 45 kV, tube current of 40 mA in a step size scan mode (0.03° min^−1^) and scanning electron microscopy images (2) with the Secondary Electron Detector at low voltage of 2 kV, 2000x of magnification and a working distance of 4.3 mm of OLV loaded γ-CD-MOF formulations using KNO_3_ (**A**) and KCl (**B**) as potassium sources

**Fig. 2:**
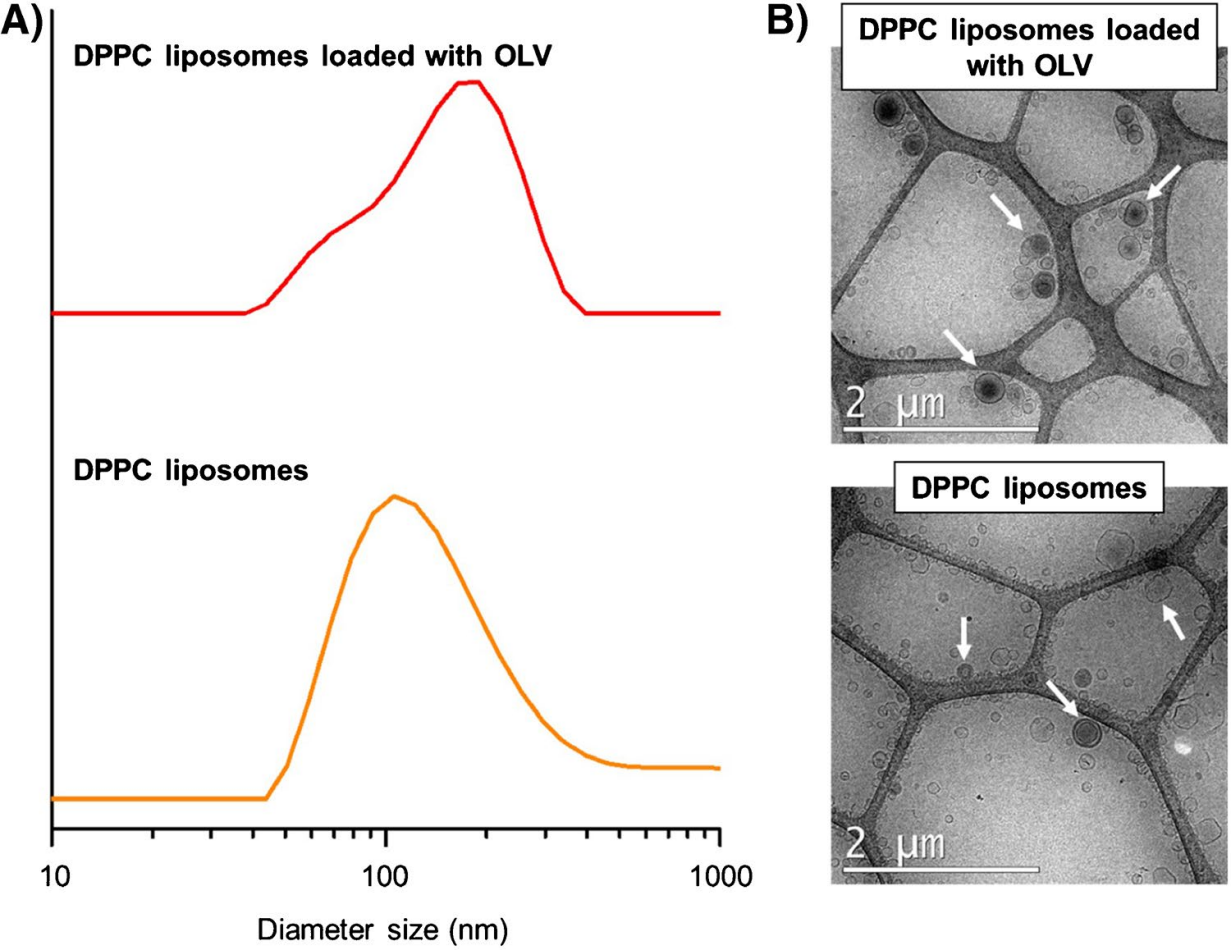
Size distribution by intensity (**A**) using a laser with *λ*_0_ = 632.8 nm, 175° angle, 1.0031 cP, and 1.330 of R.I. for the viscosity of water and cryo-TEM images (**B**) using 5000x of magnification of OLV loaded DPPC liposomes and DPPC liposomes in HEPES buffer solution

Regarding the liposomes’ characterization, the size distribution is a key aspect to consider, even more when the final application is as drug delivery systems. In order to get information about the size of DPPC liposomes and the physical stability of these systems after the encapsulation of olivetol, DLS and cryo-TEM analysis were performed. DPPC formulations loaded with the drug showed more polydispersity than empty DPPC liposomes with wider and more asymmetric peaks in DLS results (Fig. 2A). These results were in agreement with cryo-TEM analysis where bigger liposomes and with several concentric lipidic bilayers could be observed in the samples with OLV (Fig. 2B). The mean diameter of the liposomes was below 200 nm: 125 ± 52 nm for DPPC liposomes and 159 ± 67 nm for DPPC liposomes loaded with OLV. Mean diameter and standard deviation were similar in both liposomes’ formulations. However, the size distribution observed in the liposomes without drug was closer to a normal distribution than the formulations with OLV, and the polydispersity index values were lower in the empty liposomes (0.3) than in the loaded ones (0.9). This fact means that the samples with low polydispersity index had homogeneous distribution of the vesicles of similar diameters without aggregates. It could be explained since olivetol is an organic compound and it is encapsulated in the bilayer increasing the rigidity of liposomes, such as the effect of cholesterol addition [24]. Cryo-TEM analysis corroborated these results. Anyway, the synthesis method was found to be useful for producing stable DPPC liposomes with and without olivetol of relatively controllable particle size.

The UV-Vis spectrophotometry analysis confirmed the presence of olivetol in all the carriers (Table 2). For MOF formulations, OLV encapsulation percentages were in the order of the values reported in previous studies using CD-MOFs for the encapsulation of different compounds [25]. For liposome formulation, OLV encapsulation percentage was higher than the values for both MOFs. These percentages could not be compared between MOFs and liposomes, since the values are expressed in terms of mass and the weight of each carrier is different [22, 25]. In order to verify if the amount of drug applied over the teeth in liposomes and MOF formulations was comparable, the OLV amount per application was calculated based on the sample preparation process (Tooth sample preparation). This amount was of the same order for the three carriers (Table 2). Besides, the amount of drug applied without carrier as positive control was extremely high to study the efficiency when the carrier was used. In any case, the drug could be quantified in all the formulations, making them suitable as carriers for olivetol and for their application over the tooth structure.

**Table 2:** Olivetol encapsulation percentage for each formulation with their standard deviations and drug amount per application

	OLV encapsulation (%)	OLV/CBD amount per application (µg)
DPPC loaded with olivetol MLV (molar ratio 2:1)	9.1 ± 0.1	36.8 ± 0.5
γ-CD-MOFs KCl loaded with olivetol	2.4 ± 0.3	24 ± 3
γ-CD-MOFs KNO_3_ loaded with olivetol	1.6 ± 0.2	16 ± 2
CBD or OLV (10 mg/mL)	–	1000

### Peaks assignment of enamel and dentin

**Fig. 3:**
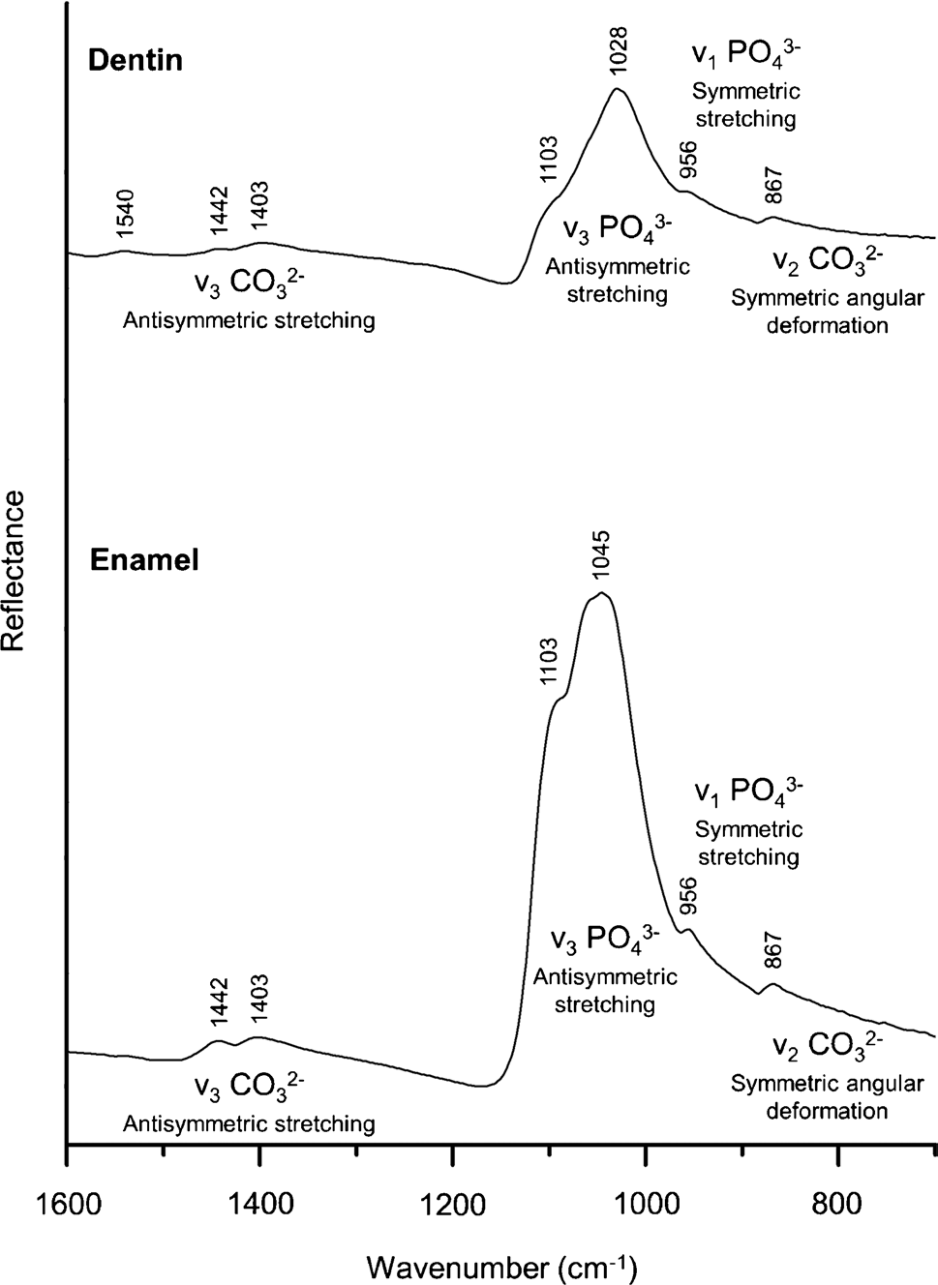
Average of the specular reflectance FTIR spectra of control enamel and dentin of bovine tooth and band assignment numbers (*n*=30). The spectra were acquired in the reflection mode and for each one, 64 scans were carried out from 4000 cm^−1^ to 600 cm^−1^ with 4 cm^−1^ spectral resolution at room temperature, using 10×10 μm^2^ of aperture size. A gold mirror was used as background reference

Enamel and dentin are the main tissues that comprise the teeth. Enamel is composed of 96% of mineral content, while in the dentin it is reduced to approximately 70%. Regarding the organic matter, the enamel presents only 2% and, in the dentin, it increases to 20%. The inorganic mineral that mainly composes both tissues is the carbonated calcium-deficient hydroxyapatite (HAP), which is a crystalline calcium phosphate [Ca_10_(PO_4_)_6_(OH)_2_] and carbonates impurities [CO_3_^2−^] [26]. Bovine enamel and dentin reflection spectra are shown in Fig. 3 between 1600 and 700 cm^−1^ where the bands of carbonate and phosphate groups of HAP could be observed. Due to the high degree of crystallinity of the enamel, the bands that correspond to this mineral matrix are more intense than in the dentin tissue which has a low crystallinity [27]. Antisymmetric stretching vibration of phosphates groups (v_3_ PO_4_^3−^) appears as a wide band in the 1200–900 cm^−1^ region, in both enamel and dentin tissues with a maximum at 1045 and 1028 cm^−1^, respectively. These broad bands are attributed to triply degenerated v_3_ PO_4_^3−^ [28]. The shoulder at 956 cm^−1^ corresponds to the symmetric stretching vibration of the same group (v_1_ PO_4_^3−^) in both tissues [29]. The peaks at 1403 and 1442 cm^−1^ match with antisymmetric stretching vibration of carbonate groups (v_3_ CO_3_^2−^) and the one at 867 cm^−1^ with symmetric angular deformation (v_2_ CO_3_^2−^) in both tissues. Besides, organic matter (amide II) is detected at 1540 cm^−1^ in the dentin, since its percentage in this tissue is higher than that in the enamel.

### Study of the treatments’ effect over the enamel

**Fig. 4:**
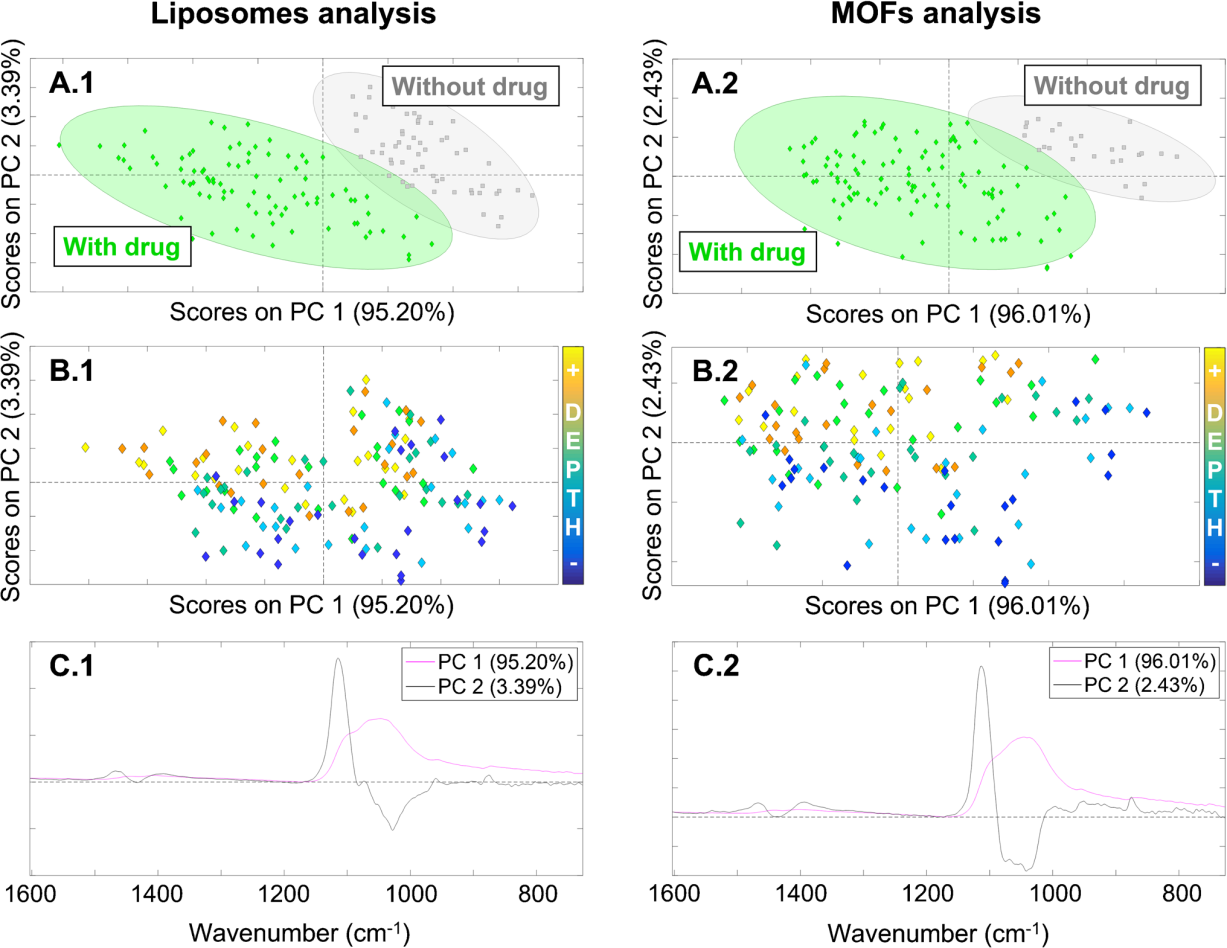
Principal component analysis of liposomes (1) and MOFs (2) in the inner enamel; a mapping of 30 spectra was analyzed per sample from 1600 to 700 cm^−1^, and mean centering was applied as preprocessing technique; score plots grouping according to the drug presence (**A**); score plots showing the distribution across the enamel depth (**B**); loading profiles of PC1 and PC2 (**C**)

In the present study, the enamel FTIR spectra of the different samples were analyzed by PCA, where the weights of the main components were concentrated in the components 1 and 2 (PC1 and PC2) for both carriers. Liposomes and MOF analysis had a PC1 of 95.20% and 96.01% versus PC2 of 3.39% and 2.43% accounting for a total of 98.59% and 98.44% of the variance, respectively (Fig. 4). The two carriers’ analysis had a similar distribution in the enamel, and only two groups could be distinguished along PC1 according to drug presence (Fig. 4A). The group with drug included CBD, OLV, and OLV-loaded liposomes or MOFs (depending on the carrier analysis), and the group without drug comprised HEPES control for both carriers and also unloaded liposomes for liposome analysis. Therefore, it revealed that both OLV-loaded carriers may transport the drug, since these treatments were in the same group as CBD and OLV control treatments. Nevertheless, the separation between the groups that contained or not drug was not perfectly clear. This fact could be explained due to the large inorganic material content and high crystallinity of the enamel [30]. Because of these enamel properties, the signal of carbonate and phosphate groups of HAP was too high overlapping the possible vibrations peaks of organic material that can be present in the enamel, as the drugs and carriers employed in the different treatments [31]. For this same reason, no more differences related with the treatments could be observed between groups, since spectra were distributed according to the depth in the enamel structure along PC2 (Fig. 4B).

Regarding the loading graphs (Fig. 4C), PC1 comprised all the bands of carbonates and phosphates groups of HAP previously assigned for both liposomes and MOF analysis in the positive part of the graphs. Taking into account the enamel structure properties, no contribution of drugs, liposome, or MOF vibration peaks were expected to be distinguished, since their signals are not significant in comparison with the signal of the inorganic material of the enamel [32]. Thus, only changes over HAP structure could be noted. The treatments with drug (CBD, OLV, OLV-loaded liposomes, and MOFs) produced the decrease of the bands of carbonate and phosphate groups of HAP in comparison with the average of the spectra of the samples without drug, especially in the antisymmetric stretching vibration v_3_ PO_4_^3−^ (Fig. S3.A). This fact could be explained due to the organic matter increment in the samples treated with drug, because the crystallinity of the enamel in these samples was worse detected than in the ones without any drug, since they have better reflective properties, and its infrared signal is higher [33]. Besides, shifts were not detected in the average of the spectra of the samples with and without drug for both carriers, so the treatments did not compromise carbonate and phosphate groups of HAP [34]. These results agreed with the distribution in scores graphs along PC1 (Fig. 4A), where the group with drug is in the negative part of the component (less intense signal) and the group without drug in the positive part (more intense signal).

PC2 loading profiles revealed in the positive part of the graphs, v_3_ CO_3_^2−^ and v_2_ CO_3_^2−^ peaks of carbonate groups from 1475 to 1380 cm^−1^ and 867 cm^−1^, respectively (Fig. 4C). A secondary phase vibration of v_3_ PO_4_^3−^ at 1113 cm^−1^ was observed. This vibration contributes to the shoulder at 1103 cm^−1^ of the peak assignment, and it is related with the bandwidth [63]. Meanwhile, the maximum peak of v_3_ PO_4_^3−^ was in the negative part. This profile was in agreement with the distribution of the scores according to the depth in the enamel structure (Fig. 4B). Enamel tissue presents heterogeneous distribution of the inorganic material, since its carbonate content increases from the outer to inner enamel and the phosphates decrease showing more crystallinity and more packing in the outer part [35]. Therefore, the fact that the scores in the positive part of the graph correspond to the inner parts of the enamel and the ones in the negative part to the outer region matched with the PC2 loading profiles for both carriers. This information is in accordance with the typical mineral gradient of the enamel, verifying that PC2 distributes the samples following the depth in this tissue.

### Study of the treatments’ effect over the dentin

**Fig. 5:**
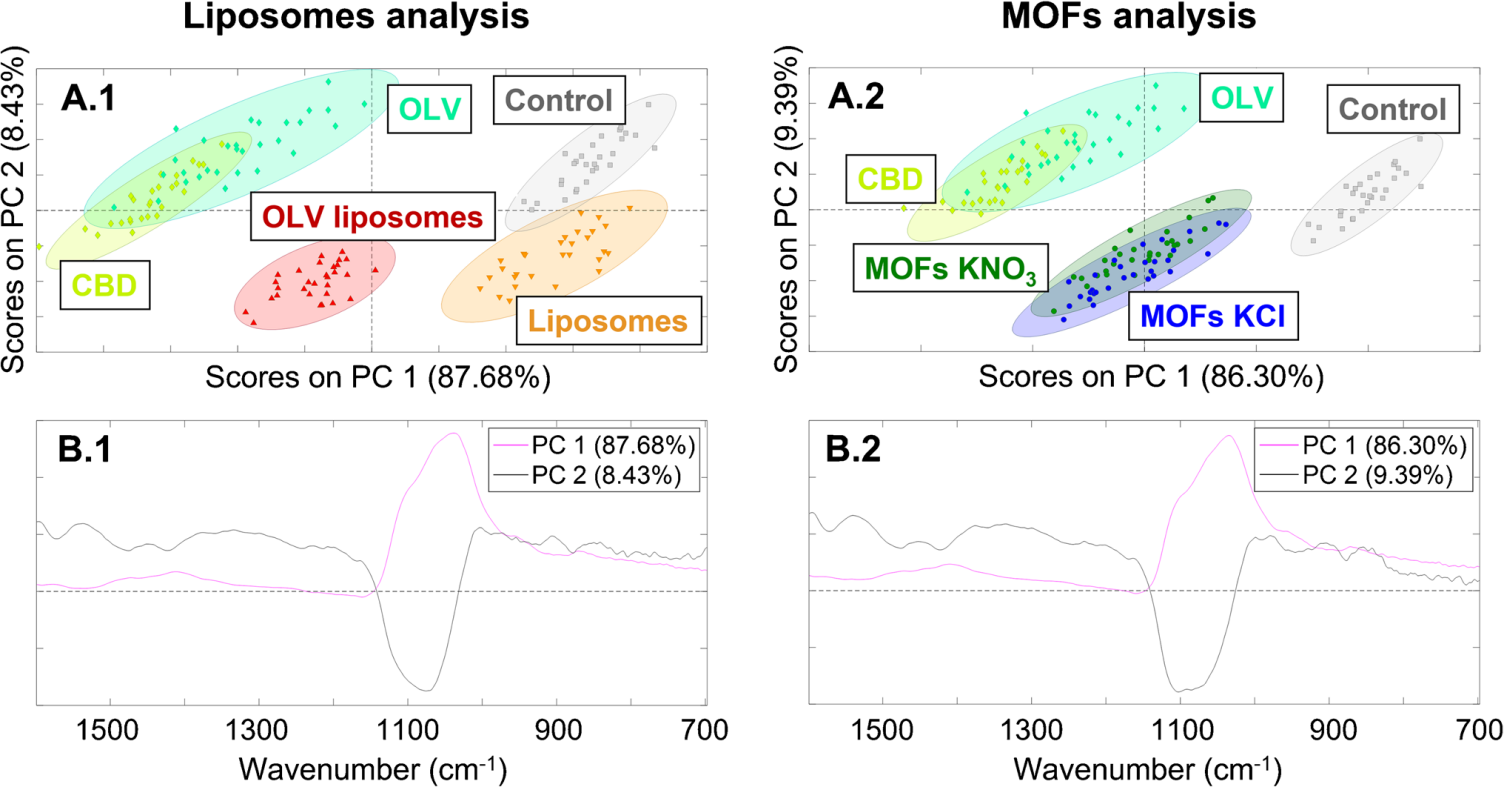
Principal component analysis of liposomes (1) and MOFs (2) in the inner dentin; a mapping of 30 spectra was analyzed per sample from 1600 to 700 cm-1 and mean centering was applied as pre-processing technique; scores plots grouping according to the drug presence and carrier presence (**A**); loadings profiles of PC1 and PC2 (**B**)

The analysis of the dentin was also performed using PCA for the different treatments. As in the enamel results, the weights of the main components were in the components 1 and 2 (PC1 and PC2) for both carriers. Liposomes and MOF analysis had a PC1 of 87.68% and 86.30% versus PC2 of 8.43% and 9.39% accounting for a total of 96.11% and 95.69% of the variance, respectively (Fig. 5A). In comparison to the enamel analysis, the separation between the groups in the dentin was much better and more consistent information can be obtained. This better separation of the different groups in the dentin may be due to the less mineral content and less crystal packing in this tissue, having the organic matter of the drug stronger presence improving the sample grouping [27]. Therefore, the signal of carbonate and phosphate groups of HAP decreases, and the vibrations of the organic material could be distinguished. All the groups in both carriers’ analysis had similar distribution in the scores’ graphs along PC1 and PC2 (Fig. 5A). Loaded liposome and MOF groups were closer to the samples treated with only drug (CBD and OLV) than the samples without treatment or unloaded carrier. Thus, it means that both carriers led the drug transport until the dentin tissue efficiently increasing the diffusion process, despite the drug concentration per application was much lower than in the CBD and OLV control groups (Table 2) [15]. Furthermore, CBD and OLV control groups are completely overlapped in this analysis, confirming that OLV could be used as CBD analog in this experiment. Dentin analysis also revealed differences related to the presence of liposomes or MOFs along PC2. Meanwhile, the groups without any carrier were in the positive part of PC2, the groups with carrier were in the negative part of this component regardless the drug presence. Therefore, PC1 may group the samples according to the drug presence and PC2 according to the carrier presence in the dentin tissue. Moreover, these results from the dentin analysis verified and supported the previous results of the enamel.

PC1 loading graphs included all the characteristic vibrations previously assigned in the dentin tissue for both carriers in the positive part of the profile (Fig. 5B). As in the enamel analysis, drug vibrations did not appear since the contribution of the inorganic material was much more significant that the contribution of the drug. The comparison between the average of the spectra without drug with the average of the spectra with drug (CBD, OLV, OLV-loaded liposomes, and MOFs) revealed a reduction in the intensity of all the bands, related to a higher organic matter content in the dentin tissue that decreased the crystallinity and the signal of the bands (Fig. S3.B) [36]. The only band that did not decrease is the one at 1540 cm^−1^ in case of the liposome analysis, since this vibration may be related to the NH_3_^+^ deformation from the DPPC structure avoiding the loss of intensity of this peak despite the decrease in crystallinity due to the drug presence [37]. However, the main vibrations did not shift, so the chemical structure of the dentin was not changed. The loading profiles of PC1 were in agreement with the scores’ graphs since the samples without drug were in the positive part of this component (more intense signal) and the samples with drug were in less positive values (less intense signal).

PC2 profiles comprised the wide band of the antisymmetric stretching vibration v_3_ PO_4_^3−^ in the negative part emphasizing the shoulder at 1103 cm^−1^ of the peaks’ assignment, while the rest of the peaks were in the positive half of this component (Fig. 5B). This negative band gives information about the bandwidth and the groups that were in the more negative part of the scores’ graphs correspond to treatments with liposomes and MOFs that contributed with the increase of the organic material in the dentin tissue, reducing the crystallinity and making the mentioned phosphate bandwidth when these groups were compared to the ones without any carrier [38]. This information agreed with score distribution along PC2 where the samples without carrier were in the positive part and the samples with carrier in the negative half. The fact that the rest of the bands were in the positive part means that the presence of any carrier did not affect these vibrations.

These results have demonstrated that thanks to the developed formulation using these two DDS, CBD can reach the dentin in an efficient way where the drug will be able to exert its analgesic effect. Without the proposed carriers, CBD could not be able to reach it due to its low chemical stability and poor aqueous solubility, unless the drug is applied in a high concentration, which would not be viable in terms of toxicity to tissues or in economic terms at the market level [39]. Even though there are a great number of possible strategies as a treatment of DH, up to date, none of them has yet been found to be efficient [40]. In any case, further insights could be performed in the development of these materials, such as the optimization of the formulations in terms of drug encapsulation to improve its efficacy. Another aspect to consider, it is the stability of the DDS in a suitable marketed formulation. For example, a liquid or gel formulation based on water would not be appropriate since the degradation will be very fast [41]. Other important point is to verify whether scaling up the production of the formulation from the laboratory level to the industrial level is technically and economically possible. Thus, this research will lead to the development of important advances in drug delivery technologies and in oral health, since it is the first time that these carriers has been used in combination of CBD against DH [42].

## Conclusions

In the present study, olivetol-loaded γ-CD-MOFs and DPPC liposomes have been proposed as potential analgesic delivery systems of CBD for DH treatment. PCA showed that the treatment could reach the enamel and the dentin as PC1 grouped the samples according to the drug presence in both tissues, meanwhile, PC2 grouped the samples according to the depth in the enamel structure due to the typical mineral gradient of this tissue. Moreover, no differences were observed between OLV and CBD validating the hypothesis of OLV application as CBD analog in this experimental approach.

On the other hand, OLV loaded liposomes and MOFs targeted the drug transport in a more efficient way, since these groups were closer to the CBD and OLV control groups despite the fact that drug concentration per application was much lower. Therefore, it was possible to trace the penetration of the drug in the tooth structure and to know that the carriers helped in the delivery process making it more efficient. Besides, shifts in the HA bands were not observed meaning that the treatments did not affect the tooth structure.

In conclusion, new potential analgesic delivery systems loaded with cannabinoids for oral health were developed, leading the drug transport in an efficient way to cross the tooth structure. Without the developed DDS, CBD will not be able to reach the dentin due to its low chemical stability and poor aqueous solubility; there are no other materials used as CBD carriers for oral health. However, further insights must be explored from the laboratory to the final market in order to assess the encapsulation capacity and stability.

## Supplementary Information

Below is the link to the electronic supplementary material.

Supplementary file1 (DOCX 397 KB)
