# Supplementary material for: Efficient controlled release of cannabinoids loaded in γ-CD-MOFs and DPPC liposomes as novel delivery systems in oral health
Source: Mikrochim Acta. 2023 Mar 9;190(4):125. doi: 10.1007/s00604-023-05692-4 (PMC9998313; doi:10.1007/s00604-023-05692-4)
Supplement: Supplementary file 1 — Supplementary file1 (DOCX 397 KB) [file 604_2023_5692_MOESM1_ESM.docx]

**Electronic Supplementary Material**

**Efficient controlled release of cannabinoids loaded in γ-CD-MOFs and DPPC Liposomes as novel delivery systems in oral health**

Jorge Rodríguez-Martínez^1(¥)^, María-Jesús Sánchez-Martín*^1(¥)^, Manuel Valiente^1^

^1^GTS Research Group, Department of Chemistry, Faculty of Science, Universitat Autònoma de Barcelona, 08193 Bellaterra, Spain.

^(¥)^ Shared co-first authorship

^*^Co-first author and author for correspondence: [mariajesus.sanchez@uab.cat](mailto:mariajesus.sanchez@uab.cat) (ORCiD: 0000-0003-1678-6055)


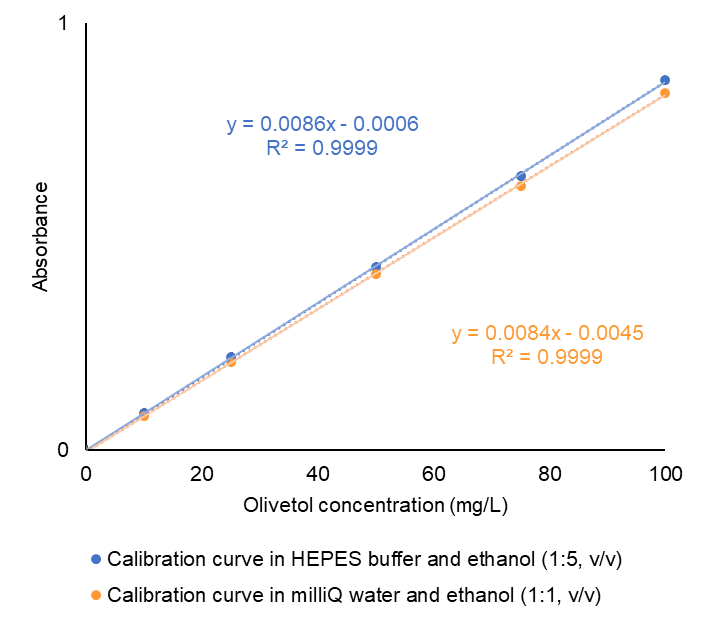


Figure S.1: Calibration curves of different known concentrations of olivetol from 0 to 100 mg/L in HEPES buffer and ethanol (1:5, v/v) (blue) and milliQ water and ethanol (1:1, v/v) (orange) media for the quantification of the drug in DPPC liposomes and MOFs, respectively.


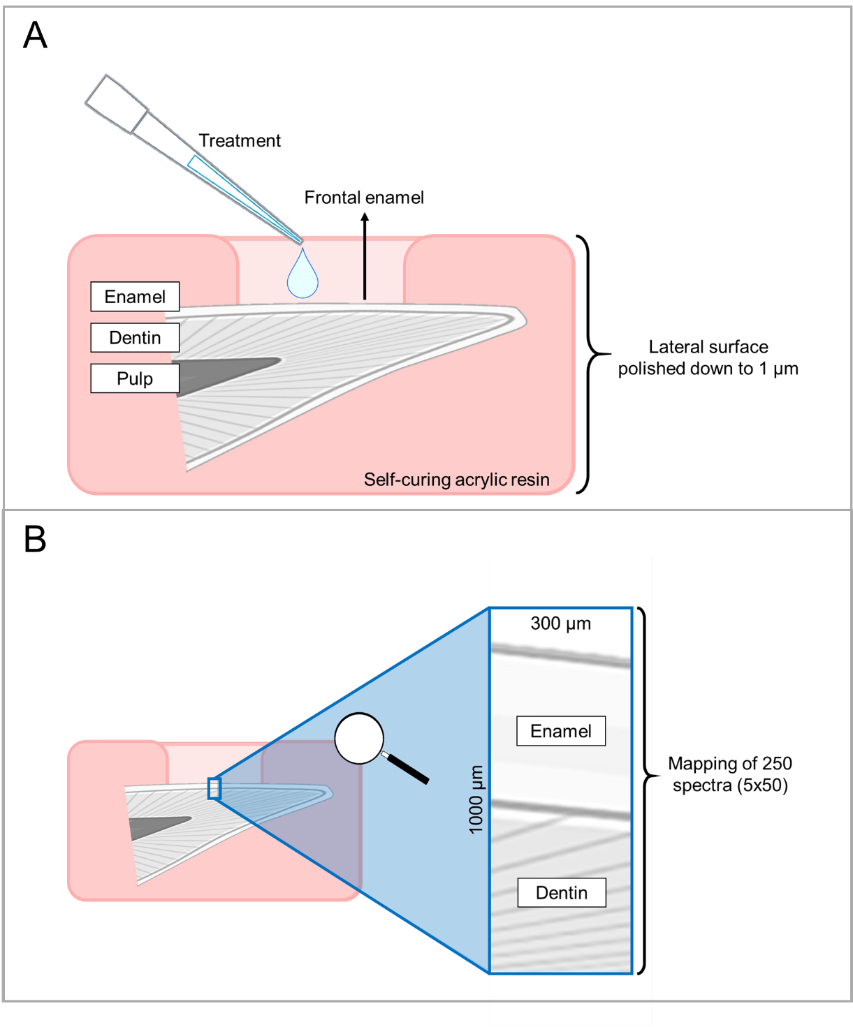


Figure S.2: Sample preparation and treatment application over the teeth diagram (A) and synchrotron source infrared spectroscopy measurements procedure (B).


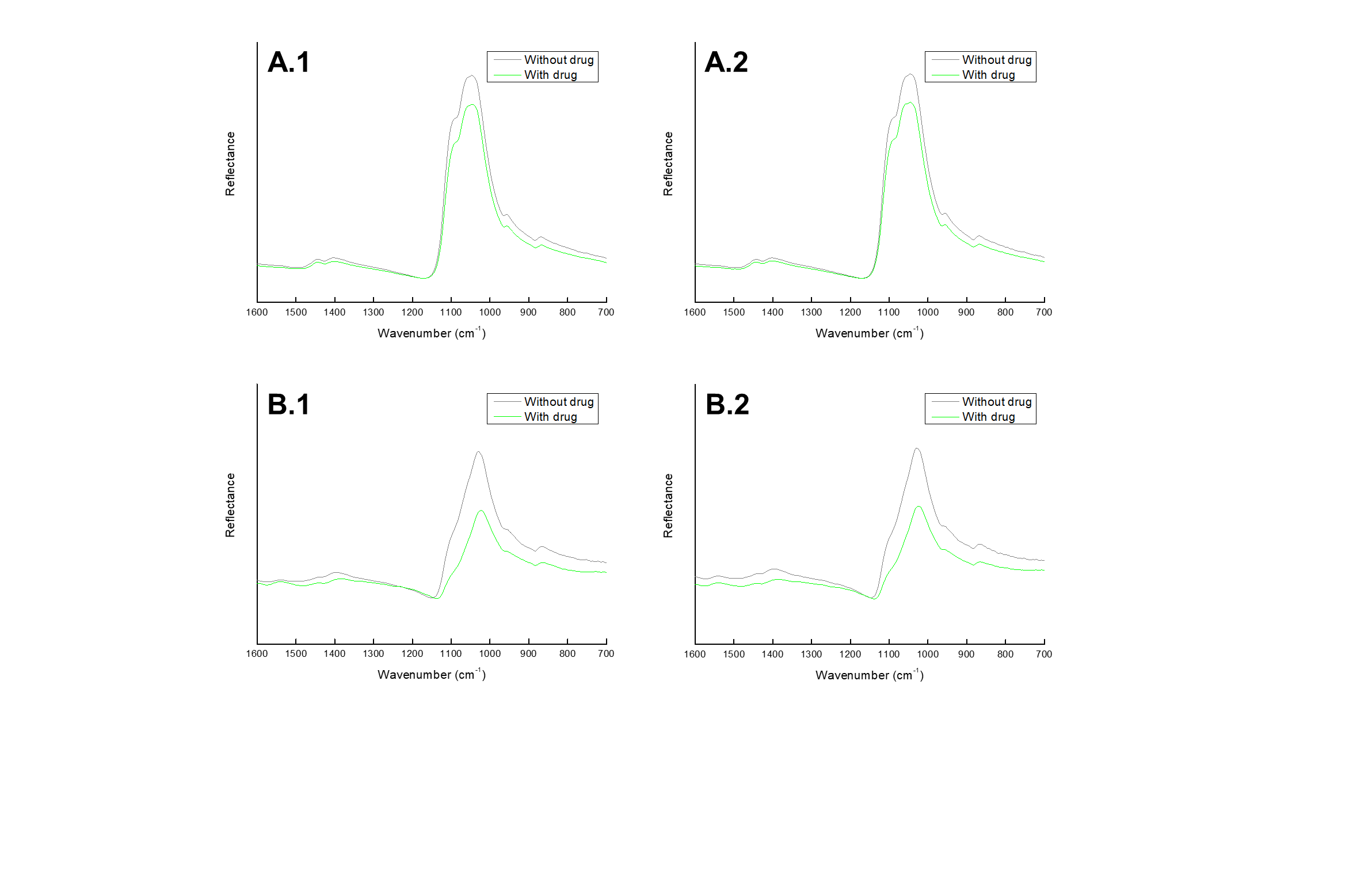


Figure S.3: Average of the spectra of the groups with and without drug for liposomes (1) and MOFs (2) in the enamel (A) and the dentin (B).
